# The Effect of Psychological First Aid Interventions on Self-Efficacy and Professional Quality of Life Among Physicians: A Quasi-Experimental Study

**DOI:** 10.3390/ejihpe15120245

**Published:** 2025-11-29

**Authors:** Othman A. Alfuqaha, Uday M. Al-Masarwah, Fatima M. Al Talahin, Rihan Thaher Altarawneh

**Affiliations:** 1 Department of Psychology, College of Humanities and Sciences, Ajman University, Ajman 346, United Arab Emirates; 2 Counseling and Mental Health Department, Faculty of Educational Sciences, The World Islamic Sciences & Education University W.I.S.E., Amman 11947, Jordan; odai.masarweh@wise.edu.jo (U.M.A.-M.); fatmeh.tlaheen@wise.edu.jo (F.M.A.T.); Rihan.tarawneh@wise.edu.jo (R.T.A.)

**Keywords:** psychological first aid, self-efficacy, professional quality of life, physicians, Jordan

## Abstract

(1) Background: Psychological first aid (PFA) interventions are designed to tackle the effects of traumatic events on individuals to help them reach stability. (2) Methods: We conducted a quasi-experimental study. A total of 162 physicians in Jordan were conveniently selected between 28 November and 15 December 2023. We identified 82 participants by a simple random procedure to represent the control group = 42 and the experimental group = 40. The PFA intervention course was initiated from 18 December 2023 to 21 February 2024. (3) Results: We found significant differences (*p* < 0.001) between the control group and the experimental group based on knowledge, skills, and attitudes in terms of PFA; self-efficacy (SE); and professional quality of life (ProQoL). The experimental group demonstrated higher knowledge, skills, and attitudes in terms of PFA and improved SE compared to the control group. Physicians, before undergoing the PFA intervention and strategy training course, exhibited moderate levels of compassion satisfaction (CS), burnout (BO), and secondary traumatic stress (STS). After the training course, both BO (M = 3.14, t = 3.44, *p* < 0.001, Cohen’s *d* = 0.56) and STS (M = 2.01, t = 4.25, *p* = 0.001, Cohen’s *d* = 0.65) decreased significantly in the experimental group, while there was no significant effect on CS (M = 4.29, t = 1.56, *p* = 0.12, Cohen’s *d* = 0.21) as a result of the PFA training course in the experimental group. (4) Conclusions: The PFA training course improves SE and ProQoL and increases knowledge, skills, and attitudes toward PFA. We recommend implementing PFA training courses for other healthcare professionals.

## 1. Introduction

Traumatic events can affect anyone and may negatively impact the physical, emotional, social, and spiritual aspects of human life. In recent years, the world has experienced an increase in traumatic events, including earthquakes, the coronavirus pandemic, chronic diseases, and wars, which have led to serious physical and psychological issues among different groups of people (Alfuqaha et al., 2023; Leppold & Reifels, 2024). The physical issues that can result from traumatic events include heart attacks, strokes, and memory problems (Thurston et al., 2023; Yang et al., 2023), while psychological issues include depression, anxiety, post-traumatic stress disorder (PTSD), and emotional/cognitive issues (Spytska, 2024; Düken et al., 2024). Thus, decision-makers and health systems should prioritize combating these traumatic events to mitigate future problems.

Physicians play a major role in the hierarchy of hospitals because of their responsibilities and tasks. It is widely recognized that they are more likely to suffer physically and emotionally compared to those in other professions (Niewiadomska et al., 2022). In clinical settings, physicians focus mainly on managing patients’ physical symptoms, but the debate continues regarding their role in supporting those with mental health issues after traumatic events (Søvold et al., 2021). In contemporary practice, mental health and well-being have significantly improved in various settings, but evaluating physicians’ preparedness to perform psychological first aid (PFA) interventions for their patients is important in the era of frequent traumatic events.

PFA interventions are designed to tackle the effect of traumatic events on individuals to reach stability (Shah et al., 2020). The goals of PFA are to alleviate stress, provide a sense of security, promote relationships, and nurture emotions (Willis et al., 2021). A systematic review study found that implementation of PFA interventions among traumatized patients enhances overall mental well-being (Hermosilla et al., 2023), reduces anxiety, and facilitates adaptation (Wang et al., 2024). While PFA interventions can be applied by specialists in various organizations, physicians in hospitals require education and training to integrate PFA interventions into patient care protocols who suffer from traumatic events such as loss, chronic diseases, and cancers. It is worth mentioning that there is a lack of studies focusing on physicians compared to other groups (Heavey, 2021; Geoffrion et al., 2023). Therefore, well-educated and trained physicians in PFA interventions can facilitate the recovery of patients. Challenges caused by traumatic events, such as COVID-19, wars, and frequent violence toward HCPs, have led practitioners and researchers to implement PFA interventions via online platforms (Alfuqaha et al., 2022; Ni et al., 2024). Implementing PFA interventions in actual settings is more effective than online settings. Thus, we hypothesized that implementing a PFA training course for physicians may have a direct effect on traumatized patients’ quality of life and mental health.

Self-efficacy (SE) plays a major role in managing traumatic situations. It serves as a protective factor against traumatic events (Hoelterhoff & Chung, 2020) and contributes effectively to mitigating adverse traumatic outcomes (Morison & Benight, 2022). Previous studies found that the higher one’s SE, the more capable one is of implementing PFA strategies (Chandra et al., 2014), increasing their quality of life, and lowering BO levels (Matos et al., 2022). Conversely, individuals with lower SE more often experience greater stressors and reduced personal commitment and motivation (Alfuqaha et al., 2019; Barni et al., 2019). A previous study found that after implementing the clinical communication skill course, physicians’ SE was significantly improved (Gulbrandsen et al., 2009). In Turkey, it was found that implementing PFA interventions improved disaster preparation and SE among nursing students (Kılıç & Şimşek, 2019). Studying the impact of PFA intervention on SE among physicians still needs more attention. Thus, we hypothesized that implementing PFA interventions can improve physicians’ SE.

Due to physicians’ experience of several issues related to patients, their quality of life can be affected. Quality of life can be defined as the holistic emotions and feelings experienced in either a positive or negative manner as part of this profession (Post, 2014). Professional quality of life (ProQoL) has three core elements, including satisfaction levels, occurrence of traumatic stressors, and BO (Alonazi et al., 2023). Several factors were found to be affected by ProQoL, including SE (Peng et al., 2022), environmental factors (Balakhdar & Alharbi, 2023), and well-being (Schoultz et al., 2022). Therefore, a healthy workplace, especially for physicians, enhances productivity (Zhenjing et al., 2022), increases satisfaction (Bahari et al., 2022), and improves communication (Penberthy et al., 2018). Thus, we hypothesized that implementing PFA interventions can improve physicians’ ProQoL.

Some studies have investigated the interplay between ProQoL, SE, and related factors. For example, a recent study among nurses reported significant associations between these variables (Al-Otaibi & Kerari, 2025). Another study examined ProQOL and its components among advocates for victims of sexual assault (Hiroyama et al., 2025). Moreover, previous studies have indicated that PFA interventions have a direct effect on BO and ProQol (Kılıç & Şimşek, 2019; Asaoka et al., 2023). However, limited studies have explored how these important variables are associated with PFA interventions, particularly among physicians.

### 1.1. Study Aims

**Figure 1:**
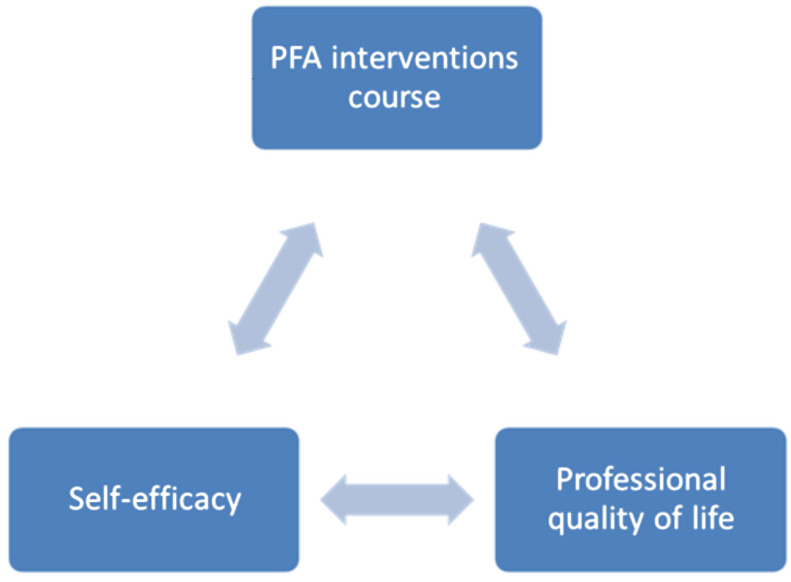
Relationships between the selected variables.

This is among the first studies in Jordan, to our knowledge, that aim to implement a PFA interventions and strategies course among physicians in hospitals. It is essential to note that there is a lack of PFA training courses among physicians in clinical settings (Iiyama et al., 2023; Dieltjens et al., 2014). Furthermore, the connection between important psychological factors such as SE and ProQoL with PFA interventions will shed light on the importance of implementing a PFA course in hospitals. Thus, we also aim to explore the effect of a PFA interventions and strategies course on SE and ProQoL among physicians in Jordan (Figure 1).

### 1.2. Study Hypothesis

**Hypothesis** **1.** *Demonstrating the PFA interventions and strategies course to physicians in hospitals will improve their knowledge, skills, and attitudes toward PFA.*

**Hypothesis** **2.** *Implementation of the PFA interventions and strategies course will improve physicians’ SE.*

**Hypothesis** **3.** *Implementation of the PFA interventions and strategies course will enhance physicians’ ProQoL.*

## 2. Materials and Methods

### 2.1. Design and Participants

We conducted a quasi-experimental study following the CONSORT guidelines (Sarveravan et al., 2017). Our target population comprised hospital-based physicians. We conveniently selected physicians employed at a hospital located in Amman, the capital of Jordan. This hospital is known for its sufficient number of physicians and accessibility to researchers. Inclusion criteria included various departments within the hospital, residents who had completed the year of privilege, and had not been enrolled previously in the PFA interventions and strategies course. Physicians who refused to participate, undergraduate students, and maternity/unpaid leave were excluded. Moreover, physicians working in psychiatric units, as they are familiar with PFA interventions and strategies, were also excluded.

### 2.2. Study Procedure

Before conducting this study, we obtained ethical approval from Jordan University Hospital (10/2023/30560). Then, we initiated the distribution of online Google Forms (28 November and 15 December 2023) via official groups of physicians and among their supervisors/managers, asking for assistance in distributing it among their physicians. The distributed form included a consent form (“Do you agree”: yes/no), demographic data (gender, marital status, age, length of experience, and educational levels), Arabic versions of scales (PFA, SE, and ProQol), and questions related to their agreement to attend the PFA interventions and strategies course, providing their phone number. The online survey required approximately 5 min to complete. A sufficient number of participants according to G-power software (Version 25) was 150 participants based on (a = 0.05, power 0.80, and medium effect size). Thus, a total of 162 physicians filled in the online questionnaire successfully.

**Figure 2:**
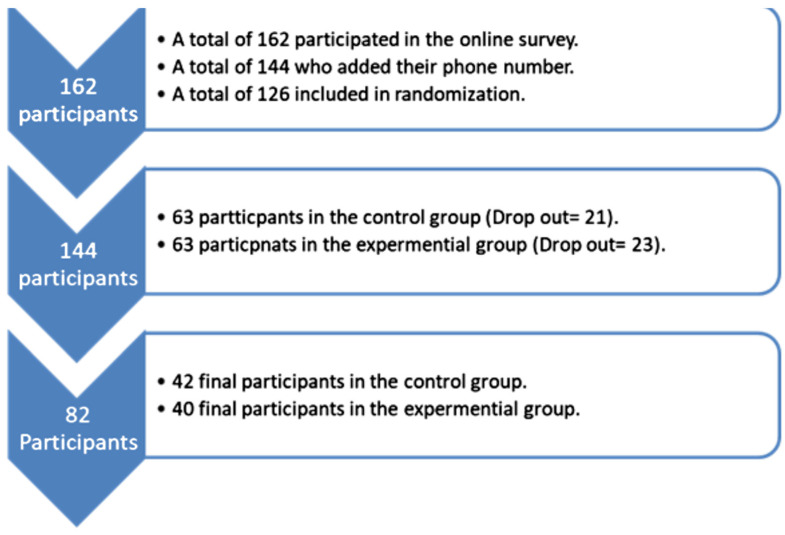
Participant recruitment flowchart.

After that, we contacted all physicians who had provided their phone numbers via phone call (*n* = 144) to explain the nature of the PFA training course. After calling, a total of 18 participants refused to participate, leaving a sample size of 126 participants. Thus, we randomly divided the sample size (n = 126) into two groups (control and experimental), with 63 participants in each, using a simple random procedure. At the first meeting, we explained the purpose of the PFA interventions course and assured them of their voluntary enrollment in this course. Regrettably, several participants dropped out due to insufficient time, work pressure, and not being interested in the PFA training course due to their specialization. Thus, the final participants amounted to 42 in the control group and 40 in the experimental group (Figure 2). That number was considered sufficient (Laktarash et al., 2019). However, the pre-test for both groups was on 17 December 2023, and the post-test was on 21 February 2024.

### 2.3. Study Measures

PFA Questionnaire: We used the Arabic validated PFA scale (Alshareef et al., 2025). This scale aims to assess three cores: knowledge, skills, and attitudes toward PFA (McCabe et al., 2014). As a self-report tool, participants provide responses based on their perceptions and experiences. It employs a five-point Likert scale ranging from “Strongly Agree” to “Strongly Disagree”. It comprises 18 items measuring knowledge (9 items), skills (7 items), and attitudes (5 items) beneficial for responding to traumatic events. A higher mean score suggests higher knowledge, skills, and attitudes toward PFA. It has been utilized in various studies, indicating its validity and reliability (Kouvatsou et al., 2022; Alshareef et al., 2025). Cronbach’s alpha values were 0.85, 0.84, and 0.81 for knowledge, skills, and attitudes toward PFA, respectively.

#### 2.3.1. PFA Interventions and Strategies Training Course

The researchers are experts in psychological and educational counseling and in conducting PFA courses. The researchers conducted all training courses in the educational center in the selected hospital. All PFA training courses follow a standardized training manual to ensure fidelity. The experimental group took the PFA training course, and the control group took a general communication skills course of similar duration and format to the PFA sessions. Attendance was recorded for each session, and participants were required to attend at least 75% of the sessions to be considered adherent to the intervention. The course started on 17 December 2023 and ended on 21 February 2024. During that period, there was a session of one hour every week, and the details of 6 sessions are illustrated as follows:

##### Session One

Acquaintance, trust relationship, general information about the PFA training course, aims, principles, and people who specifically need the PFA training course.

##### Session Two

Definitions of traumatic events, PTSD, depression, anxiety, victims, signs and symptoms of traumatized patients, causes, grief process, and finally, feedback sessions.

##### Session Three

Criteria of DSM, 5TR according to previous mental disorders in session two, cut-off standards, clinical practice to diagnose these patients, role play, group discussion, and finally, feedback sessions.

##### Session Four

Definitions of PFA and its components, including the five cores of PFA training (safety, listen, comfort, hope, connection), discussion about SE and ProQoL, role play, group discussion, and finally, feedback sessions.

##### Session Five

Skills exercise based on cognitive, behavioral, and spiritual strategies. Cognitive skills include cognitive restructuring and problem-solving. Behavioral skills include stress inoculation and coping skills. Spiritual skills include connection/support, connection with God, and self-compassion.

##### Session Six

Evaluation, improvement, administering the post-test, and finally, feedback about the PFA training course.

#### 2.3.2. General Self-Efficacy Scale

This is a universal scale assessing self-belief in challenging situations, consisting of 10 items, adapted from the study of (Schwarzer & Jerusalem, 1995). It has been translated into 32 languages, including Arabic. Respondents use a four-point Likert scale, ranging from “Exactly true” to “Not at all true,” reflecting the original scale. The Arabic validated version was used, and we calculated the Cronbach’s alpha value, which was 0.83, indicating a reliable scale. The higher the average mean scores, the higher the levels of SE.

#### 2.3.3. Professional Quality of Life Scale (ProQoL)

This scale is designed to measure the quality of life within this profession over the past 30 days. It contains three dimensions—compassion satisfaction (CS), BO, and secondary traumatic stress (STS)—with 10 items in each category (Stamm, 2009). Physicians were requested to respond on a five-point Likert scale, ranging from 5 = “Very often” to 1 = “Never”. Items 1, 4, 15, 17, and 29 ranged in reverse order. As in the original score, cut-off points lower than 22 are considered a low score; scores from 23 to 41 are considered moderate; and above 41 is considered a high score. This scale has demonstrated validity and reliability in previous studies, including within the Arabic context (Geoffrion et al., 2019; Algamdi, 2022). We measured the Cronbach’s alpha values for the Arabic scales and subscales, and they were 0.88, 0.81, and 0.91 for CS, BO, and STS, respectively. The total Cronbach alpha value was 0.80.

### 2.4. Data Analysis

Data were exported from an Excel sheet to SPSS v.23. Descriptive statistics were employed for demographic factors and main variables. Internal consistency was assessed using Cronbach’s alpha. We used Pearson’s correlation coefficient (*r*) to explain the relationships between main variables. Furthermore, we used paired sample *t*-tests to explore the differences between two groups regarding knowledge, skills, and attitudes toward PFA, SE, and ProQoL before demonstrating the PFA interventions and strategies training course. The independent sample *t*-test was used to illustrate the differences between two groups after demonstrating the PFA interventions and strategies training course. To calculate the effect size between each construct, we used Cohen’s d with values 0.2, 0.5, and 0.8 illustrating small, medium, and large effects, respectively. The significance value was set at 0.05.

## 3. Results

### 3.1. Descriptions of Participating Physicians

Among (162) physicians, more than half were male (57.4%), single (58.6%), had experience of 1 to 5 years (55.6%), and had completed their bachelor’s degree (53.1%). The average age was 26.41 ± 3.63 (Table 1).

### 3.2. Correlation Analysis of PFA Dimensions and Related Variables

The correlation analysis for 162 physicians shows positive correlations (*p* < 0.05) between all dimensions of PFA. The SE is positively associated (*p* < 0.05) with all PFA dimensions. The BO dimension is negatively associated with all the examined variables except the STS dimension, with which it is positively associated (*r* = 0.33, *p* < 0.001). The STS is not found to be associated with PFA dimensions. The STS is negatively associated with SE (*r* = −0.29, *p* < 0.001). The CS is positively associated with skills and attitudes toward PFA (*r* = 0.22, *p* < 0.001; *r* = 0.34, *p* < 0.001), respectively (Table 2).

### 3.3. Comparison Between Control and Experimental Group

The final number of participating physicians, after distributing them into control and experimental groups, was 42 and 40, respectively. The average age was 25.7 ± 3.38 in the control group, while in the experimental group, it was 27.8 ± 3.17. Male participants were more likely to be interested in the training course than females in both groups. The length of experience in the control group ranged from 1 to 5 years, while in the experimental group, it ranged from 6 to 10 years (Table 3).

Among 162 participants, the total mean scores were 2.83, 3.14, and 3.34 for knowledge, skills, and attitudes toward PFA, respectively. SE exhibited a mean score of (3.06 ± 0.37). The sum scores of ProQoL dimensions were 40.3, 34.9, and 28 for CS, BO, and STS, respectively. Based on the cut-off values, these scores were considered at a moderate level. We also calculated the confidence intervals, effect sizes, and mean scores of ProQoL dimensions, and the results are demonstrated in Table 4.

Among the control (n = 42) and experimental group (n = 40), and before demonstrating the PFA interventions and strategies training course, we ran a paired sample *t*-test. The results showed that there were no differences (*p* > 0.05) between the two groups based on PFA, SE, and ProQoL dimensions.

After demonstrating the PFA interventions and strategies training course, we ran an independent samples *t*-test between both groups. The results showed that the experimental group demonstrated higher levels of all PFA dimensions, more SE, and lower BO and STS with a large effect size, and the differences were statistically significant (*p* < 0.001) compared to the control group. There were no significant differences between the experimental group and the control group based on the CS dimension of ProQoL, and the effect was found to be low (*p* = 0.12, *d* = 0.21) (Table 4).

## 4. Discussion

This study highlights significant differences between the control and experimental groups, which underwent the PFA interventions and strategies training course, based on PFA knowledge, skills, and attitudes. The experimental group demonstrated greater knowledge, skills, and attitudes toward PFA compared to the control group. Additionally, this study shows that physicians, before demonstrating the PFA interventions and strategies training course, exhibited moderate levels of CS, BO, and STS. After demonstrating the PFA training course, both BO and STS constructs of ProQol decreased significantly among physicians in the experimental group compared to the control group. Furthermore, SE increased significantly after demonstrating the PFA training course among physicians in the experimental group compared to the control group. Finally, we found positive correlations between all PFA dimensions and SE and CS among all participating physicians.

A previous cross-sectional study measured the levels of PFA knowledge, skills, and attitudes without any training course. They found that 39% of participants had a moderate level of PFA knowledge, 51.2% practiced PFA at a moderate level, and 42.3% had a positive attitude (Zakaria & Chuemchit, 2017). Compared to our results, PFA knowledge was relatively low before demonstrating the PFA training course, while after demonstrating the PFA interventions and strategies training course, participants exhibited higher levels of knowledge, skills, and positive attitudes toward PFA. This course provides in-depth information about what traumatized patients experience during crises and teaches specific skills to deal with suffering patients, such as reflecting emotions and active listening. As a result, physicians are capable of dealing with such events. This result aligns with a previous study conducted in Canada, which also found an improvement in PFA knowledge and skills after a training course (Lalani & Drolet, 2018). A review of 23 studies was conducted to examine the PFA training application. They found that the PFA training significantly improves PFA knowledge, skills, and attitudes in supporting individuals in acute distress (Wang et al., 2021).

We found positive correlations between PFA constructs and both SE and CS. In other words, the higher the knowledge, skills, and attitudes of PFA, the higher the SE and CS. Physicians who have experience dealing with PFA are more likely to have resilience (Kunzler et al., 2020) and a sense of awareness and accomplishment (Hung et al., 2019). On the other hand, negative correlations were found between PFA constructs, SE, CS, and BO. Physicians who have high knowledge and skills and positive attitudes toward PFA can manage their emotions, mitigate stress levels, and have greater confidence and competence. This study is consistent with a study conducted in Japan, which found greater BO levels among physicians who did not attend the PFA training course (Asaoka et al., 2023).

In our study, physicians perceived their ProQoL (CS, BO, STS) as being at moderate levels before completing the PFA interventions and strategies training course. They perceived moderate levels of BO and STS signs and symptoms. Our results are closely linked with those of a previous study, which found that 78.9% of participants had high BO, 76% had moderate potential CS, and 82% had moderate potential compassion fatigue among 167 physicians (Abdelhadi Ibrahim et al., 2021). But after completing the PFA interventions and strategies training course, the experimental group had less BO and STS compared to the control group, while there was no difference between the two groups based on CS. Surprisingly, in 2020, a study found no effect of the PFA training course on ProQol among HCPs (Sijbrandij et al., 2020).

Our PFA interventions and strategies training course has a direct effect on both SE and ProQoL, but does not affect CS. Based on SE, we believe that practical skills, including role play, and taught skills based on cognitive, behavioral, and spiritual approaches, as well as group discussions, improve self-confidence and trust, which enables physicians to deal with traumatized patients effectively. Taught skills include cognitive restructuring, stress inoculation, coping skills, connection/support, and self-compassion (Abdelhadi Ibrahim et al., 2021). This finding is consistent with a previous study, which found that PFA training courses significantly enhance HCPs’ SE (Everly & Lating, 2022). On the other hand, a study found that the PFA training course did not affect HCPs’ SE (Sijbrandij et al., 2020). Based on the ProQoL, we also believe that our PFA training course mitigates feelings of BO and STS, improves coping strategies, and enhances the overall well-being of physicians. This study can be useful to alleviate BO among HCPs by applying the PFA training course across various professions, such as nurses (Alfuqaha & Alsharah, 2018). In previous studies, they found that a PFA training course reduces PTSD and depressive symptoms (Figueroa et al., 2024), prevents suicide (Hernandez-Cervantes, 2021), and decreases traumatic incidents in organizations (Lewis et al., 2014). Regarding CS, we found that the PFA training course had no effect. This may be related to the higher scores observed before implementing the PFA sessions or because CS is a relatively stable professional trait. Thus, we recommend that more psychological factors and longer follow-up periods should be studied with the PFA training course.

Our study’s findings could be considered strengths, particularly with the lack of previous studies that investigated the effect of PFA interventions and strategies training courses among physicians in hospitals. Focusing on psychological factors such as SE and ProQoL provides information about such training courses. We recommend expanding this idea from hospital settings to the community by involving psychologists, counselors, or anyone interested in PFA to raise public awareness about its importance during crises, as mentioned in a recent study that tackled Italian people affected by both COVID-19 and an earthquake (Perilli et al., 2025).

However, these findings are limited by several factors, including its small sample, single-site design, convenience sampling, self-reported questionnaires, attrition bias, not being pre-registered, and lack of long-term follow-up. Future studies must address these limitations.

## 5. Conclusions

We found significant differences between the control group and the experimental group in terms of knowledge, skills, and attitudes toward PFA, SE, and ProQoL. The experimental group demonstrated more SE, better knowledge and skills, and more positive attitudes toward PFA compared to the control group. Physicians, before completing the PFA interventions and strategies training course, exhibited moderate levels of CS, BO, and STS, while after the training PFA course, both BO and STS decreased significantly in the experimental group. Finally, we found positive correlations between all PFA dimensions and SE and CS. Even so, this study has several limitations that warrant consideration in future studies. Thus, we recommend implementing the PFA training course for other healthcare professionals and conducting longitudinal studies to assess the long-term effects of the PFA training course on SE and ProQoL. We also recommend that researchers and practitioners implement PFA interventions and strategies interested in psychological and educational interventions to enhance their practical value in crises and traumatic events.

## Figures and Tables

**Table 1:** Demographic information of physicians (n = 162).

Variables	Descriptive	Frequency (%)
Gender	Male	93 (57.4)
	Female	69 (42.6)
Marital status	Single	95 (58.6)
	Married	65 (40.2)
	Widow/divorced	2 (1.2)
Age	Years (M ± SD)	26.41 ± 3.63
Educational levels	Bachelor’s degree	86 (53.1)
	Master’s degree	74 (45.7)
	PhD degree	2 (1.2)
Length of experience	1–5 years	90 (55.6)
	6–10 years	56 (34.5)
	Higher than 10 years	16 (9.9)

Note. M ± SD: mean ± standard deviation.

**Table 2:** Correlation coefficient for the examined variables (n = 162).

%	Variables	1	2	3	4	5	6	7
1	Knowledge of PFA	1.00	0.66 ** 1.00	0.51 **	0.13 *	0.03	−0.20 *	0.10
2	Skills of PFA			0.67 **	0.26 **	0.22 **	−0.23 **	0.09
3	Attitudes of PFA			1.00	0.20 *	0.34 **	−0.33 **	0.08
4	Self-efficacy				1.00	0.61 **	−0.23 **	−0.29 **
5	CS					1.00	−0.41 ** 1.00	−0.36 **
6	Burnout							0.33 **
7	STS							1.00

Note. PFA: psychological first aid, CS: compassion satisfaction, STS: secondary traumatic stress, M ± SD: mean ± standard deviation, * *p* < 0.05, ** *p* < 0.001.

**Table 3:** Demographic information among control (n = 42) and experimental group (n = 40) of physicians.

Variables	Descriptive	Control Group n (%)	Experimental Group n (%)
Gender	Male	22 (52.4%)	30 (75%)
	Female	20 (47.6%)	10 (25)
Marital status	Single	26 (61.9%)	21 (52.5%)
	Married	16 (38.1%)	19 (47.5%)
Age	Years (M ± SD)	25.7 ± 3.38	27.8 ± 3.17
Educational levels	Bachelor’s degree	24 (57.1%)	20 (50%)
	Master’s degree	18 (42.9%)	20 (50%)
Length of experience	1–5 years	23 (54.8%)	12 (30%)
	6–10 years	16 (38.1%)	20 (50%)
	Higher than 10 years	3 (7.1%)	8 (20%)

Note: M ± SD: mean ± standard deviation.

**Table 4:** Comparison between experimental and control groups before and after PFA training course.

Variables	All Participants (n = 162) M ± SD	Pre-Intervention		Post-Intervention		Independent Sample *t*-Test	*p*-Value	Cohen’s *d*	95% CI
		Control (n = 42)	Exp (n = 40)	Control (n = 42)	Exp (n = 40)				
Knowledge Skills Attitudes of PFA	2.83 ± 0.60	2.82 ± 0.58	2.85 ± 0.61	2.98 ± 0.59	4.01 ± 0.64	7.58	<0.001 **	0.66	0.33–1.10
	3.14 ± 0.61	3.12 ± 0.63	3.15 ± 0.59	3.16 ± 0.61	4.21 ± 0.59	7.92	<0.001 **	0.71	0.44–1.26
	3.34 ± 0.61	3.33 ± 0.52	3.35 ± 0.67	3.43 ± 0.54	4.15 ± 0.62	5.62	<0.001 **	0.51	0.13–0.88
Self-efficacy	3.06 ± 0.37	3.08 ± 0.38	3.04 ± 0.39	3.15 ± 0.33	3.56 ± 0.40	4.07	<0.001 **	0.56	0.19–0.93
CS Burnout STS	4.03 ± 0.56	4.05 ± 0.55	4.01 ± 0.56	4.10 ± 0.53	4.29 ± 0.54	1.56	0.12	0.21	−0.14–0.53
	3.49 ± 0.37	3.45 ± 0.39	3.51 ± 0.42	3.40 ± 0.37	3.14 ± 0.31	3.44	<0.001 **	0.56	0.18–0.95
	2.80 ± 0.81	2.83 ± 0.77	2.78 ± 0.65	2.71 ± 0.75	2.01 ± 0.74	4.25	<0.001 **	0.65	0.31–0.91

Note. PFA: psychological first aid, CS: compassion satisfaction, STS: secondary traumatic stress, Exp: experimental group, M ± SD: mean ± standard deviation, CI: confidence interval. ** *p*-value < 0.001.

## Data Availability

The data that support the findings of this study are available on request from the corresponding author. The data are not publicly available due to privacy or ethical restrictions in Jordan University Hospital. For data requests please contact the following email: juhosp@ju.edu.jo.

## Abbreviations

The following abbreviations are used in this manuscript:

BO	Burnout
PFA	Psychological First Aids
ProQol	Professional Quality of Life
SE	Self-efficacy
STS	Secondary Traumatic Stress
